# Learning and Development of Diagnostic Reasoning in Occupational Therapy Undergraduate Students

**DOI:** 10.1155/2020/6934579

**Published:** 2020-08-19

**Authors:** Pedro Moruno-Miralles, Adriana Reyes-Torres, Miguel-Ángel Talavera-Valverde, Ana-Isabel Souto-Gómez, Luis-Javier Márquez-Álvarez

**Affiliations:** ^1^Department of Nursing, Physiotherapy and Occupational Therapy, University of Castilla-La Mancha, Talavera de la Reina, Toledo, Spain; ^2^Health Department, Faculty Human Rehabilitation, University of the Valle, Cali, Colombia; ^3^Integra Saúde Research Unit, Health Science Department, Faculty of Health Science, Universidade da Coruña, Campus de A Coruña, A Coruña, Spain; ^4^University School of Social Work, Universidade Santiago de Compostela, Santiago de Compostela, Spain; ^5^Faculty Padre Ossó, University of Oviedo, Oviedo, Spain

## Abstract

**Background/Aim:**

One way to facilitate occupational therapy undergraduate students transferring their academic skills of data gathering and analysis to professional settings is to ensure they can competently use diagnostic reasoning. Nevertheless, there are several obvious gaps in empirical evidence related to the learning and development of this style of reasoning in occupational therapy undergraduates. The most important are related to promoting higher-order thinking and the use of information to solve problems in the context of professional practice. This study analyses undergraduates' diagnostic reasoning and its changes during their education.

**Materials and Methods:**

This multicentre study was conducted with a descriptive observational design. The study took place at the University of Coruña (Spain), University of Castilla-La Mancha (Spain), and University of el Valle (Colombia). The sample was *n* = 247. For data collection, a clinical case was specifically designed. IBM SPSS Statistics (v19) and EPIDAT 3.1 were used for the data analysis.

**Results:**

Participants identified and categorized occupational performance problems. However, they had difficulties when identifying and categorizing the occupational performance components (specifically, the symptoms and signs of the disease presented in the study case). They presented limitations to analyse and synthesize the information collected to develop an explanation of the occupational problems and their causes.

**Conclusions:**

Undergraduate students' ability to analyse and synthesize information during data collection is poorly organized, so it makes the problem formulation difficult. This study contributes to the knowledge of undergraduates' diagnostic reasoning features, specifically the undergraduate students' capacities and limits to process information during the occupational assessment.

## 1. Introduction

Encouraged by the studies on diagnostic reasoning done by the discipline of medicine since the early 1980s, the study of professional reasoning in occupational therapy has led to several lines of research [1]. One of the most important lines is the study of the information processing involved in diagnostic reasoning understood as a process of problem solving or decision making [2–7]. This area of research has provided ways to look at the competencies that occupational therapists need to handle information and arrive at an occupational therapy diagnosis in different practice fields [8–12].

Diagnostic reasoning examines and analyses cause(s) or nature of conditions requiring occupational therapy intervention and attempts to explain why a client is experiencing problems using a blend of scientific-based and client-based information [13]. When diagnostic reasoning is not well constructed, the causes of performance deficits could be misidentified, which could lead to the incorrect intervention principles being followed and thus lead to ineffective treatment [14].

According to Rogers and Holm [6], the problem-solving process that leads to the occupational therapy diagnosis is referred to as diagnostic reasoning. The occupational diagnosis usually consists of components: descriptive, explanatory, cue, and pathologic [6] (Table 1).

Thus, the occupational therapy diagnosis is the product of diagnostic reasoning, the result of the problem-solving process during the initial assessment.

The information processing involved in diagnostic reasoning [10, 15, 16] implies two processes. The first is to acquire cues and recognise patterns during data collection to identify information on occupational performance areas, symptoms and signs, skills, performance patterns, and characteristics of the environment (hereafter, components of performance). This information is categorized thanks to a preselected theoretical framework of reference. The second is to formulate the problem, which allows analysing and synthesizing the information collected in an occupational therapy diagnosis to develop an explanation of the occupational problems and their causes. In this process, not only do experienced occupational therapists have a great deal more knowledge than novices but also their knowledge is more diverse, better organized, and in a more accessible way due to related prior experience [17]. Robertson [18] and Robertson et al. [19] looked at differences between novices and experts in occupational therapists' professional reasoning and found that undergraduate students and experts had access to the same information. However, the information was more clearly defined and highly organized by the experts who develop knowledge networks that are reinforced by working with similar cases over time. This repetition is essential for the development of useful knowledge networks and cannot be replicated by exercises in academic environments.

In the case of undergraduate students, the information was less defined than in the case of the experts due to the lack to domain-specific knowledge related to problem representation. Accordingly, the way in which the novices organize their knowledge to analyse and synthesize the information gathered during the initial assessment is an element of primary importance to acquire a proper professional reasoning during their academic education.

In occupational therapy, the line of research that focuses on the study of the differences between novices vs. experts in professional reasoning has been extensive [1, 20, 21]. However, there are several obvious gaps in empirical evidence, “but ones of such importance that they bear highlighting” are those related to information processing, fieldwork supervision, and personal variances in reasoning [22]. Particularly, those related to the learning and development of diagnostic reasoning of undergraduate students of occupational therapy (no specific references in the literature have been found for this topic). According to Farber and Koenig [23] for the future of the profession, “we need to strive toward facilitating the students' best clinical reasoning and to promote relevant problem-solving strategies.” As a result, to know the characteristics of the undergraduates' diagnostic reasoning is a prime objective.

From our point of view, although the body of knowledge on clinical reasoning in OT is extensive, it is still inadequate [24]. According with Schaaf [25], Rochmawati and Wiechula [26], and Bondoc [27], educators should start examining their practices and aligning those practices with the best evidence concerning instructional methods. We consider that this purpose fully justifies this research. The overall objective of this study is to analyse undergraduates' diagnostic reasoning and its changes during their education. The specific objectives are (a) to analyse the undergraduates' ability in identifying and categorizing information during data collection, (b) to analyse the undergraduates' ability in analysing and synthesizing information for problem formulation, and (c) to analyse the changes done during the undergraduates' education.

## 2. Materials and Methods

### 2.1. Ethics

The study obtained the approval of the University of Valle Human Ethics Review Institutional Committee (ethics approval number VRI/1772); the Galician Network of Research Ethics Committees, attached to the General Technical Secretariat of the Department of Health (ethics approval number 2014/399); and the Healthcare Service of Castilla-La Mancha Clinical Research Ethics Committee (ethics approval number 2014/036).

### 2.2. Study Design

**Figure 1:**
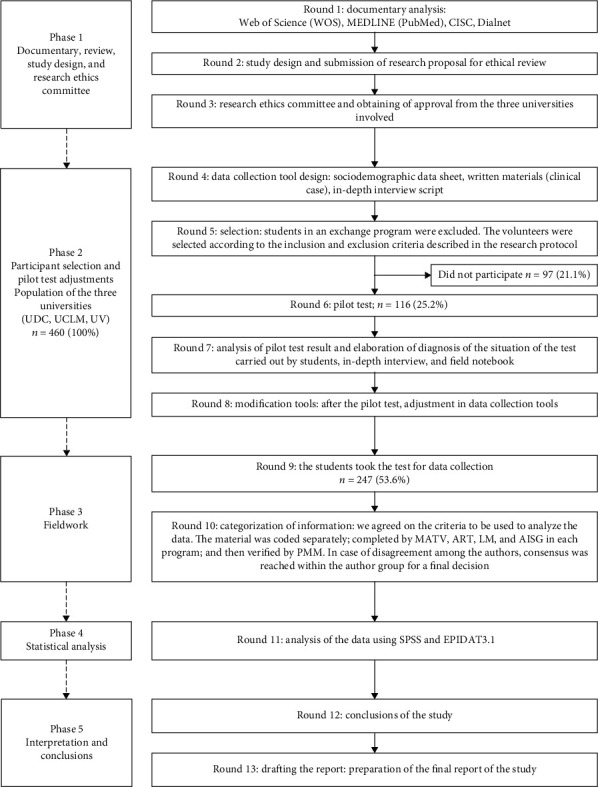
Flow chart of research stages and participants in the study.

Multicentre study [28] was conducted in five phases (from October 2013 to September 2016) and involved three universities: the University of Coruña (Spain) (UDC), University of Castilla-La Mancha (Spain) (UCLM), and University of el Valle (Colombia) (UV). This study falls within the field of educational research [29]. It was conducted with a quantitative approach and a cross-sectional study design [28] (Figure 1).

### 2.3. Participants

After reviewing the bibliography, designing the study, getting approval by the Research Ethics Committees, and obtaining the authorization from the dean of each faculty (Figure 1, phase 1), the process to select the participants started. A meeting was organized with the undergraduates in three different programs: 53 students attended at the Universidad del Valle, 90 at the Universidad de A Coruña, and 104 at the Universidad de Castilla-La Mancha, in which research goals were explained and their voluntary participation in the study was requested. Those who agreed to participate signed informed consent (IC) forms. The universities' population of the three universities in the first, third, and fourth years was 460 undergraduates, with a female predominance *n* = 392 (85.2%).

Programs length is four years. Content and teaching strategies in the three universities are similar and ordered in the same way. The curricula in all institutions comply with standards from ENOTHE and WOFT [30], and their teacher continuing education criterion and teaching strategies are common and shared by the three universities. Professional reasoning is taught using lectures, reading, problem-based learning, tutorials, and case methods. These strategies are used in specific practice fields: mental health, geriatrics, paediatrics, physical and intellectual impairments, community, and education. The three institutions were actively engaged in the study. The principal researchers are professors from all three institutions, and participants were included from all university programs.

In the selection process, first-, third-, and fourth-year undergraduates were included. The second-year undergraduates were not selected because the contents of the syllabus from the first and second years are similar concerning professional reasoning skill learning. With the aim of contextualizing the findings of this study, the structure of occupational therapy programs is described as follows. Therefore, it will be possible to identify when the different elements of professional reasoning are taught and the students' level of expertise. The students acquire generic competencies associated with basic general knowledge of professional reasoning during the two first years of education, at the three universities (Table 2).

The basic assumption was that the undergraduate students will improve their ability to identify, categorize, analyse, and synthesize the information during their education. Those who were also enrolled in a related degree and those who were retaking subjects from previous academic years were excluded. Finally, after doing the pilot test and after excluding those undergraduates that voluntarily decided not to participate, the study sample was *n* = 247 participants, with an average age of 21 ± 1.5 and a female prevalence of *n* = 222 (89.9%) (Figure 1, phase 2).

### 2.4. Data Collection

For data collection, a clinical reasoning case study was specifically designed [28] which consisted of a description of the gathered data, step by step, during the initial evaluation process. According to Neistadt et al. [31], a clinical reasoning case study is a type of case method that “illustrate the occupational therapist's thought processes by providing specific client information.” This type of case study chunks client information the way an experienced therapist might. Therefore, a case study method is reliable to assess professional reasoning to the extent that it “uses a variety of reasoning skills critical to solving real clinical problems” [32].

**Figure 2:**
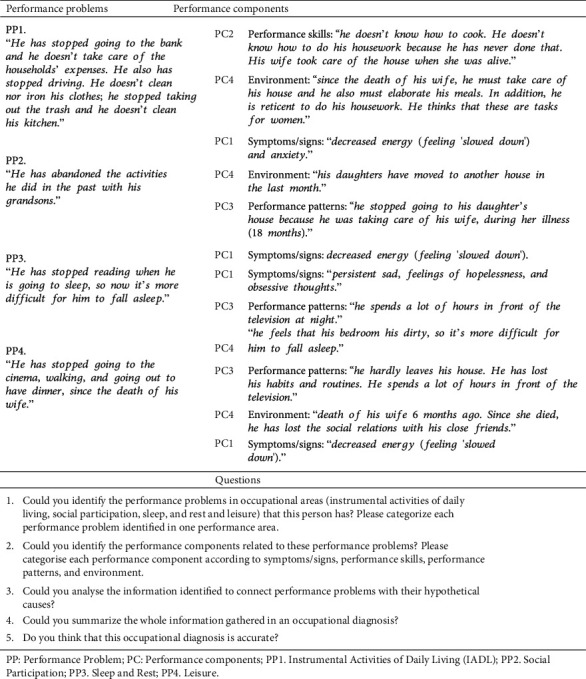
Case study. PP: performance problem; PC: performance components; PP1: instrumental activities of daily living (IADL); PP2: social participation; PP3: sleep and rest; PP4: leisure.

This case study was validated during two years with a group of occupational therapists (*n* = 150) who had more than five years of experience in different fields of practice, with a reliability coefficient of 0.98 (rxx′ = 0.98). Furthermore, it was conducted as a pilot study with students from two universities (UDC and UCLM). In this pilot study, we had 149 students from the first, second, third, and fourth academic years. The validity in the pilot study with the student's population achieved a reliability coefficient of 0.80 (rxx′ = 0.80) [28] (Figure 1, phase 2). The questions were formulated after reviewing the literature on similar studies [33]. All the questions at the end of the Figure 2 were checked by three experts (from each university), to make them easier to understand for the students. This consultation resulted in a further modification of the information contained in the case. So, it was culturally more valid for students at Universidad del Valle (Colombia). Finally, five open questions were developed for each participant to answer (Figure 2).

According to the previous questions, the participants were requested (a) to read the case in order to identify and categorize the occupational performance problems, (b) to identify and categorize the components of performance associated with the identified occupational performance problems, and (c) to formulate the problem, according to an occupational therapy diagnosis. Open questions were used to obtain the data related with the study variables, basing on the analysis of the student's answers, as described in Table 3. Each participant received the same instructions before solving the case. The test was conducted in a single room, which was large enough to keep participants working individually in small groups of five undergraduate students. After presenting the study, the authors distributed the booklets to the participants. The participants worked with the same booklet throughout the test. Time and progress throughout the sequence of steps were controlled. The case resolution time was about 60 minutes.

### 2.5. Data Analysis

The established variables for the assessment of the undergraduates' answers about the case resolution are found in Table 3.

A descriptive study of the variables registered in the study was carried out. The variables were expressed by measures of frequency and percentage. The chi-square test was used to test the null hypothesis of equality of proportions, with a confidence interval of 95%. The analysis was carried out using IBM SPSS Statistics (v19) and EPIDAT 3.1.

## 3. Results

The percentage of participants that identified and categorized information related to performance components was significantly lower than the percentage of participants that identified and categorized occupational performance problems. It should be noted that the lowest percentage was symptoms and signs (Table 4).

Regarding the knowledge organization of the undergraduates for analysing and synthesizing information for problem formulation, most participants in the study did not associate any symptoms or signs as a probable cause of performance problems. Only 4.9% of the participants associated one symptom or sign, and only three participants in their fourth year associated two or more symptoms or signs. In addition, the percentage of participants that associated only one component of performance as a probable cause of performance problems was higher than the percentage of participants that associated two or more components of performance. In short, the ability of students to analyse information was poor (Table 5).

In relation to synthesizing information in an occupational therapy diagnosis, the results show that none of the undergraduate students made a complete occupational diagnosis. Nevertheless, less than half of the participants made a partial occupational diagnosis. In this partial diagnosis, 16% identified only one variable to explain the performance problems described whereas 27% identified two or more variables (three variables were expected for a totally correct response). In summary, students showed very low percentages of occupational therapy diagnosis.

Regarding the changes during the undergraduates' education, there was a significant increase in the identification of performance problems in the IADL and social participation areas (*p* = 0.005) between academic years (hereafter, AY) 1 and 4. The increase in the identification of occupational performance problems in the leisure area was constant throughout academic education (*p* = 0.001). The comparison of the other academic years did not reveal any significant differences. With respect to the development in categorization of the identified performance problems, when AY1 vs. AY4 and AY3 vs. AY4 are compared, a significant increase in all the occupational performance areas can be identified (Table 6).

An increase in the identification of the performance components was also identified, specifically in the signs and symptoms, in the performance patterns, and in the environment (*p* = 0.001) when comparing the first and fourth academic years. Also, when the first and third academic years were compared, a significant increase in the categorization of the performance components was identified, specifically in the signs and symptoms (*p* = 0.004) and in the performance patterns (*p* = 0.001) (Table 6).

In summary, a significant improvement was identified in the identification and categorization of information into performance areas and performance components (symptoms/signs, performance patterns, and environment) throughout the students' academic education.

On the other hand, a statistically significant improvement in analysing information throughout the students' education was found in symptoms/signs and performance patterns (*p* = 0.001) and environment (*p* = 0.004), as well as an improvement in synthesizing information using an occupational therapy diagnosis. The comparison between the academic years AY1 vs. AY4 and AY3 vs. AY4 yielded a chi-square of *p* = 0.001 (Table 6).

## 4. Discussions

The results of this research show that participants identified and categorized occupational performance problems. However, they had difficulties when identifying and categorizing the occupational performance components (specifically, the symptoms and signs of the disease presented in the study case). In addition, they showed poor capacity in analysing and synthesizing the information collected to outline the problem formulation. As a result, undergraduate students have difficulty processing the occupational performance components during the initial evaluation to articulate a satisfactory explanation of the occupational problems presented in the case.

The previous considerations arise from the two findings that were obtained. The first of them is related to the undergraduate's knowledge in identifying and categorizing information during data collection. According to the literature review, undergraduate students of occupational therapy historically have difficulties in identifying and describing problems in occupational performance. However, they do not have those difficulties when it comes to describing medical and psychosocial conditions [3, 6]. Contrary to what might be expected from the literature review [34, 35], a high percentage of the participants in this research were able to identify the information related to the performance areas presented in the case study. However, they found it more difficult to identify the signs, symptoms, skills, patterns, and environmental characteristics related to such performance problems. They also found it difficult to categorize them according to a theoretical reference framework or practice model.

Although various studies [36, 37] emphasize the primacy of psychological and medical models in the education of undergraduates, this finding obtained from the study participants is contrary to that thesis. This fact may be related to (a) the strengthening of practice models in our discipline, which are focused on the study of performance and occupational participation and which are organized around the evaluation of the areas of occupational performance, and (b) the weakening of education in basic knowledge of medicine and psychology [38].

This interpretation is confirmed when the facility of participants to identify and categorize problems in performance areas is compared with the facility to identify signs, symptoms, skills, patterns, and characteristics of the environment. The latter was significantly lower, particularly in the case of pathological conditions. The percentage of participants who identified the signs and symptoms of the disease presented in the case was minimal. These facts question central aspects of occupational therapy undergraduates' education since it is a health science, and therefore, the knowledge of pathological conditions and their influence on occupational performance and participation are decisive. Strengthening our practice models should not lead to underestimate medical and psychological knowledge because they assist in the elaboration of an occupational therapy diagnosis [39].

In this regard, we advocate for reinforcing the acquisition of knowledge related to medical and psychological conditions and for consolidating the undergraduate students' education to identify and categorize this kind of data collected during the evaluation.

In the second finding, although an improvement of the knowledge organization of the undergraduates for problem formulation is identified, it is found that undergraduate students present limitations in analysing and synthesizing the information collected in order to develop an explanation of the occupational problems.

On the one hand, none of the participants elaborated a complete occupational diagnosis. In addition, although a small number of participants made a partial occupational diagnosis, this is characterized by relating a single variable to explain the identified performance problems. It seems that despite finding improvements in the clinical reasoning of students when they increase their experience and advance in their courses, this improvement is not reflected in their ability to synthesize information collected during the initial assessment. The analysis of more than one variable to explain the causes of performance problems is very low among the participants. There is a predominance of reductive explanations, focusing on performance patterns and skills as the only causes of performance problems at the expense of explanations examining the complex and changing dynamics of factors that affect occupational performance and participation [40]. In other words, they tend to make reductive interpretations of performance problems instead of doing multifactorial interpretations, which would be more aligned with the theoretical assumptions of our discipline.

This study confirms the participants' limitations in organizing their knowledge according to an occupational therapy diagnosis. As they have these difficulties, they rely on other areas of knowledge, such as medicine and psychology to elaborate an occupational therapy diagnosis [37].

Previous research findings emphasize the importance of carrying out a problem formulation, encompassed by an occupational therapy diagnosis [18, 41]. Thus, an occupational therapy diagnosis makes easier to understand the complex and dynamic interaction among symptoms/signs, performance skills, performance patterns, and environments, along with the activity demands of the occupation being performed [38]. Therefore, the causes of performance problems can be identified better, leading to the correct intervention principles being followed and thus leading to effective intervention.

The findings of this research show that participants had difficulties to elaborate an occupational therapy diagnosis. This limitation might be related to the reductive interpretation of occupational performance problems. So, interpretation moves away from the ontological exegesis of the performance problems and occupational participation that define our discipline [42].

The participants' interpretation is based fundamentally on medical and psychological variables derived from a health exegesis of individual nature. It is the opposite of other broader interpretations that relate the health of individuals, groups, and communities to environmental, social, and cultural factors. These interpretations argue that the conditions of injustice, deprivation, alienation, imbalance, and occupational apartheid have determinant effects on the capacity for performance and occupational participation of individuals, groups, and communities [43].

The difficulty to elaborate an occupational therapy diagnosis could create a gap in the understanding of the new theoretical contributions in occupational therapy.

We strongly advocate promoting the organization of knowledge brought by occupational therapy diagnosis. Strengthening the undergraduate students' education to reason professionally is the best way to progress our knowledge.

An overview of our results shows a progression in the ability of students to identify, categorize, and analyse case information. When we compared responses between first- and fourth-year students, first- and third-year students, and third- and fourth-year students, we found that the higher the year, the better is their identification of relevant information and the information analysis is more complete and diverse [41]. This finding might be explained by students increasing experience and learning who are developing more knowledge and diverse knowledge. However, results do not show a similar progression in student's ability to elaborate an occupational diagnosis. This last fact might indicate that despite the education received, students have difficulties to synthesize information, because of their difficulties to represent the problem [18, 19, 23]. Probably, this difficulty is related with the fact that the students do not have their knowledge organized well because they do not have prior experience. It would be necessary to strengthen the learning of the occupational therapy diagnosis.

In conclusion, the findings of this research with respect to undergraduate students' knowledge related to the identification and categorization of information, as well as its analysis and synthetisation, question the undergraduate's ability to carry out efficient diagnostic reasoning. This fact could lead to the incorrect intervention principles being followed and thus lead to ineffective treatment.

### 4.1. Study Limitations

Due to the characteristics of the research design, it was not possible to establish control procedures for potential confounding variables to avoid potential bias in results. However, the study does make it possible to establish the possible relationship between the variables involved to conduct analytical studies.

### 4.2. Future Lines of Research

Our results suggest reinforcing students' professional reasoning learning by improving theoretical knowledge and practical skills related to problem representation and occupational diagnosis. It should be noted that research on undergraduates' professional reasoning from English-speaking countries predominates. This suggests that this research area in developing countries has not been sufficiently studied. Therefore, further research should be undertaken to analyse in depth the cultural relevance of knowledge involved in the undergraduates' learning process of professional reasoning.

## 5. Conclusions

Undergraduate students' ability to analyse and synthesize information during data collection is poorly organized, so it makes the problem formulation difficult. Although an improvement was observed in the knowledge and its organization throughout education, the limitations on processing information when making an occupational therapy diagnosis are evident.

This study contributes to the knowledge of undergraduates' diagnostic reasoning features, specifically the undergraduate students' capacities and limits to process information during the occupational assessment.

In addition, it may have implications for the education of occupational therapy undergraduates in (a) the modification of the curricula contents to promote the knowledge related to diagnostic reasoning, (b) the consolidation of the skills to analyse and synthesize the information collected during the assessment, and (c) the promotion of the learning of an occupational therapy diagnosis scheme as the best way to consolidate the professional reasoning. A key message from the study is that an inadequate organization of the undergraduate's knowledge to process the data collected during the occupational evaluation can make the learning of an appropriate diagnostic reasoning difficult.

## Figures and Tables

**Table 1:** Structural components of occupational therapy diagnosis [6].

*Descriptive component*:
Describes the deficit in occupational status. This component reflects a problem in task performance.
For example: “unable to implement the directions on the package in order to bake a frozen potpie.”
*Explanatory component*:
This indicates the therapist's hypothesis about the probable cause of the performance problems. The explanatory component is a critical feature of the occupational diagnosis because intervention strategies vary according to presumed explanatory factors.
For example: the therapist might reason that short-term memory deficit accounts for the problem in meal preparation (more than one explanation may be given for the task dysfunction).
*Cues*:
Identifies the cues that led the therapist to conclude that there was a functional deficit and to hypothesize about the nature of the deficit.
For example: signs and symptoms or cues gathered during a meal preparation task indicative of short-term memory deficit might include “reads oven temperature setting aloud three times, but does not locate the oven dial or set the temperature.”
*Pathologic*:
Identifies the pathologic agent causing the deficit. It provides intervention parameters based on the course of the pathology, prognosis, and contraindications and guidelines for occupational performance.
For example: short-term memory deficit was a consequence of depression rather than of head trauma or presenile dementia, then problem resolution would differ.

**Table 2:** The clusters of specific competences associated with occupational therapy process and professional reasoning at the different universities.

	UDC			UCLM			UV		
	1^st^/2^nd^	3^rd^	4^th^	1^st^/2^nd^	3^rd^	4^th^	1^st^/2^nd^	3^rd^	4^th^
TFound, model, and MB	X			X			X		
PD and PF		X			X			X	X
Community and other fields			X			X		X	X
Mental health and geriatrics		X			X	X		X	X

PD and PF: physical dysfunction and paediatric fields; TFound, model, and MB: theoretical foundations, models of practice, and methodological bases.

**Table 3:** Data analysis.

To evaluate the undergraduates' knowledge related to identification and categorization of performance problems (PP), the established variables were:		
*Variables*	*Identification*	*Categorization*
PP1: IADL PP2: social participation PP3: sleep and rest PP4: leisure	One point per variable when students were able to identify each occupational problem presented.	One point per variable when students describe the specific activity related with the identified problem and name it properly in the corresponding performance area.
To evaluate the undergraduates' knowledge related to identification and categorization of performance components (PC), the established variables were:		
*Variables*	*Identification*	*Categorization*
PC1: symptoms/signs PC2: performance skills PC3: performance patterns PC4: environment	One point per variable when students were able to identify the performance components related with the performance problem.	One point per variable when students correctly name the performance components identified.
To evaluate the knowledge organization of the undergraduates of analysing information (AI), the established variables were:		
*Variables* AI1: symptoms/signs AI2: performance skills AI3: performance patterns AI4: environment	(0) For those undergraduates that were not able to associate any variable with the identified performance problems. (1) For those undergraduates that associated just one variable presented in the case with the identified performance problems. (2) For those undergraduates that were able to associate two or more variables presented in the case with the identified performance problems.	
To evaluate the knowledge organization of the undergraduates in synthesizing information, the established variables were:		
*Complete occupational diagnosis*	*Partial occupational diagnosis*	
Students were given one point for a complete diagnosis when the undergraduate identified the 4 problems in the occupational performance and was able to associate them with ≥2 performance components.	Students were given one point for a partial occupational diagnosis when the undergraduate described at least ≥1 occupational problem and was able to associate them with at least ≥1 performance component.	

**Table 4:** Results regarding identifying and categorizing information^∗^.

Performance problems and performance components		
	*n* (%)	CI (95%)
*Identification of occupational performance problems*		
PP1: instrumental activities of daily living	206 (83.4)	78.6-88.2
PP2: social participation	206 (83.4)	78.6-88.2
PP3: rest and sleep	195 (78.9)	73.7-84.2
PP4: leisure	85 (34.4)	28.3-40.5
*Identification of occupational performance components*		
PC1: symptoms/signs	15 (6.1)	2.9-9.3
PC2: performance skills	56 (22.7)	17.2-28.1
PC3: performance pattern	41 (16.6)	11.8-21.4
PC4: environment	79 (32)	8.6-17.3
*Categorization of performance problems*		
PP1: instrumental activities of daily living	72 (29.1)	23.3-35
PP2: social participation	31 (12.6)	8.2-16.9
PP3: rest and sleep	121 (49.1)	42.6-55.4
PP4: leisure	40 (16.2)	11.4-21
*Categorization of components of occupational performance*		
PC1: symptoms/signs	37 (15)	10.3-19.6
PC2: performance skills	56 (22.7)	17.2-28.1
PC3: performance pattern	42 (17)	12.1-21.9
PC4: environment	90 (36.4)	30.2-42.6

^∗^Results for the group of participants from the three universities (UV, UDC, and UCLM). Percentages are based on the total sample (*n* = 247). CI: confidence interval; PP: performance problem; PC: occupational performance components.

**Table 5:** Results regarding analysing information^∗^.

Problem formulation: analysing information						
		AY1 (*n* = 96) *n* (%)	AY3 (*n* = 85) *n* (%)	AY4 (*n* = 66) *n* (%)		Total
					*p* value^∗∗^	*n* (%)
AI1: symptoms/signs					*0.001*	
	0	96 (38.9)	83 (33.6)	53 (21.5)		232 (93.9)
	1	0 (0)	2 (0.8)	10 (4)		12 (4.9)
	≥2	0 (0)	0 (0)	3 (1.2)		3 (1.2)
AI2: performance skills					0.168	
	0	80 (32.4)	64 (25.9)	47 (19)		191 (77.3)
	1	14 (5.7)	20 (8.1)	15 (6.1)		49 (19.8)
	≥2	2 (0.8)	1 (0.4)	4 (1.6)		7 (2.8)
AI3: performance patterns					*0.001*	
	0	95 (38.5)	67 (27.1)	40 (16.2)		202 (81.8)
	1	1 (0.4)	16 (6.5)	16 (6.5)		33 (13.4)
	≥2	0 (0)	2 (0.8)	6 (2.4)		8 (3.2)
AI4: environment					*0.004*	
	0	77 (31.2)	56 (22.7)	31 (12.6)		164 (66.4)
	1	16 (6.5)	16 (6.5)	16 (6.5)		48 (19.4)
	≥2	3 (1.2)	13 (5.3)	15 (6.1)		31 (12.6)

^∗^Results for the group of participants from the three universities (UV, UDC, and UCLM). AY: academic year. Percentages are based on the total sample (*n* = 247). ^∗∗^Statistically significant variables (*p* < 0.005) are highlighted in italics.

**Table 6:** Comparison of differences among academic years: identification and categorization of problems/components of occupational performance^∗^.

Performance problems and performance components						
	AY1 (*n* = 96) *n* (%)	AY3 (*n* = 85) *n* (%)	AY4 (*n* = 66) *n* (%)			
				*p* value^∗∗^	*p* value^∗∗^	*p* value^∗∗^
				AY1 vs. AY3	AY1 vs. AY4	AY3 vs. AY4
Identification of occupational performance problems						
PP1: IADL	73 (76)	71 (83.5)	62 (93.9)	0.288	0.005	0.088
PP2: SP	73 (76)	71 (83.5)	62 (93.9)	0.052	0.005	0.088
PP3: RS	73 (76)	66 (77.6)	56 (84.8)	0.937	0.242	0.365
PP4: leisure	1 (1)	28 (32.9)	56 (84.8)	*0.001*	*0.001*	*0.001*
Identification of occupational performance components						
AI1: S/S	0 (0)	2 (0.8)	13 (5.2)	0.424	*0.001*	*0.001*
AI2: PS	16 (6.5)	21 (8.5)	19 (7.7)	0.249	0.009	0.706
AI3: PP	1 (0.4)	18 (7.3)	22 (8.9)	*0.001*	*0.001*	0.135
AI4: environment	19 (7.7)	29 (11.8)	31 (12.6)	0.044	*0.001*	0.152
Categorization of performance problems						
PP1: IADL	24 (25)	10 (11.8)	38 (57.6)	0.037	*0.001*	*0.001*
PP2: SP	4 (4.2)	6 (7.1)	38 (31.8)	0.600	*0.001*	*0.002*
PP3: RS	23 (24)	41 (482)	57 (86.4)	0.001	*0.001*	*0.001*
PP4: leisure	1 (1)	4 (4.7)	35 (53)	0.295	*0.001*	*0.001*
Categorization of components of occupational performance						
AI1: S/S	3 (3.1)	14 (16.5)	20 (30.3)	*0.004*	0.964	0.068
AI2: PS	16 (16.7)	21 (24.7)	19 (28.8)	0.248	0.099	0.705
AI3: PP	1 (1)	18 (21.2)	23 (34.8)	*0.001*	*0.001*	0.091
AI4: environment	19 (19.8)	33 (38.8)	38 (57.6)	0.007	*0.001*	0.033

Comparison and estimation of differences among academic years in the identification and categorization of each area and components of occupational performance. ^∗^Results for the group of participants from the three universities (UV, UDC, and UCLM). AY: academic year. Percentages are based on the total number of participants in each academic year. S/S: symptoms/signs; PS: performance skills; IADL: instrumental activities of daily living; SP: social participation; RS: rest and sleep; PP: performance patterns. ^∗∗^Statistically significant variables (*p* < 0.005) are highlighted in italics.

## Data Availability

The data used and/or analysed during the current study are available from the corresponding author on reasonable request.

## References

[1] Unsworth C. A. (2009). The Clinical reasoning of novice and expert occupational therapists. *Scandinavian Journal of Occupational Therapy*.

[2] Cohn E. S. (1991). Clinical reasoning: explicating complexity. *American Journal of Occupational Therapy*.

[3] Fleming M. H. (1991). Clinical reasoning in medicine compared with clinical reasoning in occupational therapy. *American Journal of Occupational Therapy*.

[4] Harries P., Tomlinson C., Notley E., Davies M., Gilhooly K. (2012). Effectiveness of a decision-training aid on referral prioritization capacity: a randomized controlled trial. *Medical Decision Making*.

[5] Rassafiani M., Ziviani J., Rodger S., Dalgleish L. (2009). Identification of occupational therapy clinical expertise: decision-making characteristics. *Australian Occupational Therapy Journal*.

[6] Rogers J. C., Holm M. B. (1991). Occupational therapy diagnostic reasoning: a component of clinical reasoning. *American Journal of Occupational Therapy*.

[7] Schell B. A., Cervero R. M. (1993). Clinical reasoning in occupational therapy: an integrative review. *American Journal of Occupational Therapy*.

[8] Doyle S. D., Bennett S., Dudgeon B. J. (2014). Sensory impairment after stroke: Exploring therapists’ clinical decision making. *Canadian Journal of Occupational Therapy*.

[9] Faller P., Hunt J., Van Hooydonk E., Mailloux Z., Schaaf R. (2016). Application of data-driven decision making using Ayres Sensory Integration®With a child with autism. *American Journal of Occupational Therapy*.

[10] Kristensen H. K., Borg T., Hounsgaard L. (2010). Aspects affecting occupational therapists' reasoning when implementing research-based evidence in stroke rehabilitation. *Scandinavian Journal of Occupational Therapy*.

[11] Kuipers K., Grice J. W. (2009). Clinical reasoning in neurology: use of the repertory grid technique to investigate the reasoning of an experienced occupational therapist. *Australian Occupational Therapy Journal*.

[12] Robertson L. J. (2016). Clinical reasoning, part 1: the nature of problem solving, a literature review. *British Journal of Occupational Therapy*.

[13] Schell B. A. B., Schell J. W., Schell B. A. B., Schell J. W. (2008). Professional reasoning as the basis of practice. *Clinical and Professional Reasoning in Occupational Therapy*.

[14] Tomlin G. S., Schell B. A. B., Schell J. W. (2008). Scientific reasoning. *Clinical and Professional Reasoning in Occupational Therapy*.

[15] Higgs J., Jones M. A., Higgs J., Jones M., Loftus S., Christensen N. (2008). Clinical decision making and multiple problem spaces. *Clinical reasoning in the health professions*.

[16] Taylor B., Robertson D., Wiratunga N., Craw S., Mitchell D., Stewart E. (2007). Using computer aided case based reasoning to support clinical reasoning in community occupational therapy. *Computer Methods and Programs in Biomedicine*.

[17] Royce C. S., Hayes M. M., Schwartzstein R. M. (2019). Teaching critical thinking: a case for instruction in cognitive biases to reduce diagnostic errors and improve patient safety. *Academic Medicine*.

[18] Robertson L. J. (2016). Clinical reasoning, part 2: novice/expert differences. *British Journal of Occupational Therapy*.

[19] Robertson D., Warrender F., Barnard S. (2015). The critical occupational therapy practitioner: how to define expertise?. *Australian Occupational Therapy Journal*.

[20] Chapparo C., Ranka J., Higgs J., Gail J., Loftus S., Christensen N. (2018). Clinical reasoning in occupational therapy. *Clinical Reasoning in the Health Professions, (4^th^ Ed)*.

[21] Unsworth C., Baker A. (2016). A systematic review of professional reasoning literature in occupational therapy. *British Journal of Occupational Therapy*.

[22] Schell B. A. B., Unsworth C. A., Schell J. W., Schell B. A. B., Schell J. W. (2008). Theory and practice: new directions for research in professional reasoning. *Clinical and Professional Reasoning in Occupational Therapy*.

[23] Farber R. S., Koenig K. P., Schell B. A. B., Schell J. W. (2008). Facilitating clinical reasoning in fieldwork: the relational context of the supervisor and student. *Clinical and Professional Reasoning in Occupational Therapy*.

[24] Hooper B., Schell B. A. B., Schell J. W. (2008). Therapists’ assumptions as a dimension of professional reasoning. *Clinical and professional reasoning in occupational therapy*.

[25] Schaaf R. C. (2015). Creating evidence for practice using data-driven decision making. *American Journal of Occupational Therapy*.

[26] Rochmawati E., Wiechula R. (2010). Education strategies to foster health professional students’ clinical reasoning skills. *Nursing and Health Sciences*.

[27] Bondoc S. (2005). Occupational therapy and evidence-based education. *Education Special Interest Section Quarterly*.

[28] Hernández R., Fernández C., Baptista M. P., Hernández R., Fernández C., Baptista M. P. (2018). Concepción o elección del diseño de investigación. *Metodología de la investigación cualitativa, (5^th^ ed)*.

[29] Sabariego M. (2004). La investigación educativa: génesis, evolución y características. *Metodología de la investigación educativa*.

[30] The Tuning Occupational Therapy Project Group (2008). *Reference Points for the Design and Delivery of Degree Programmes in Occupational Therapy*.

[31] Neistadt M. E., Wight J., Mulligan S. E. (1998). Clinical reasoning case studies as teaching tools. *American Journal of Occupational Therapy*.

[32] VanLeit B. (1995). Using the case method to develop clinical reasoning skills in problem-based learning. *American Journal of Occupational Therapy*.

[33] Lysaght R., Bent M. (2005). A comparative analysis of case presentation modalities used in clinical reasoning coursework in occupational therapy. *American Journal of Occupational Therapy*.

[34] Adam K., Peters S., Chipchase L. (2013). Knowledge, skills and professional behaviours required by occupational therapist and physiotherapist beginning practitioners in work-related practice: a systematic review. *Australian Occupational Therapy Journal*.

[35] Ikiugu M. N., Smallfield S. (2015). Instructing occupational therapy students in use of theory to guide practice. *Occupational Therapy In Health Care*.

[36] Gibson D., Velde B., Hoff T., Kvashay D., Manross P. L., Moreau V. (2000). Clinical reasoning of a novice versus an experienced occupational therapist: a qualitative study. *Occupational Therapy In Health Care*.

[37] Wong S. R., Fisher G. (2015). Comparing and using occupation-focused models. *Occupational Therapy In Health Care*.

[38] Yancosek K. E., Howell D. Integrating the dynamical systems theory, the task-oriented approach, and the practice framework for clinical reasoning. *Occupational Therapy In Health Care*.

[39] Hooper B., Atler K., Wood W. (2011). Strengths and limitations of the occupational therapy model curriculum guide as illustrated in a comprehensive curriculum revision process. *Occupational Therapy In Health Care*.

[40] American Occupational Therapy Association (2014). Occupational therapy practice framework: domain and process (3^rd^ Edition). *American Journal of Occupational Therapy*.

[41] Kuipers K., Grice J. W. (2009). The structure of novice and expert occupational therapists’ clinical reasoning before and after exposure to a domain-specific protocol. *Australian Occupational Therapy Journal*.

[42] Wilcock A. A., Wilcock A. A., Hocking C. (2015). Occupation, environment, and community development. *An occupational perspective on health (3^rd^ ed)*.

[43] Hocking C. (2017). Occupational justice as social justice: the moral claim for inclusion. *Journal of Occupational Science*.

